# Populations of a cyprinid fish are self-sustaining despite widespread feminization of males

**DOI:** 10.1186/1741-7007-12-1

**Published:** 2014-01-13

**Authors:** Patrick B Hamilton, Elizabeth Nicol, Eliane SR De-Bastos, Richard J Williams, John P Sumpter, Susan Jobling, Jamie R Stevens, Charles R Tyler

**Affiliations:** 1 Biosciences, College of Life and Environmental Sciences, University of Exeter, Exeter EX4 4QD, UK; 2 Institute for the Environment, Brunel University, Uxbridge, Middlesex UB8 3PH, UK; 3 Centre for Ecology and Hydrology, Oxfordshire OX10 8BB, UK

**Keywords:** Fishery, Genetic diversity, DNA microsatellites, Waste water treatment work, Ecotoxicology

## Abstract

**Background:**

Treated effluents from wastewater treatment works can comprise a large proportion of the flow of rivers in the developed world. Exposure to these effluents, or the steroidal estrogens they contain, feminizes wild male fish and can reduce their reproductive fitness. Long-term experimental exposures have resulted in skewed sex ratios, reproductive failures in breeding colonies, and population collapse. This suggests that environmental estrogens could threaten the sustainability of wild fish populations.

**Results:**

Here we tested this hypothesis by examining population genetic structures and effective population sizes (*N*_e_) of wild roach (*Rutilus rutilus* L.) living in English rivers contaminated with estrogenic effluents. *N*_e_ was estimated from DNA microsatellite genotypes using approximate Bayesian computation and sibling assignment methods. We found no significant negative correlation between *N*_e_ and the predicted estrogen exposure at 28 sample sites. Furthermore, examination of the population genetic structure of roach in the region showed that some populations have been confined to stretches of river with a high proportion of estrogenic effluent for multiple generations and have survived, apparently without reliance on immigration of fish from less polluted sites.

**Conclusions:**

These results demonstrate that roach populations living in some effluent-contaminated river stretches, where feminization is widespread, are self-sustaining. Although we found no evidence to suggest that exposure to estrogenic effluents is a significant driving factor in determining the size of roach breeding populations, a reduction in *N*_e_ of up to 65% is still possible for the most contaminated sites because of the wide confidence intervals associated with the statistical model.

## Background

Approximately two-thirds of the world’s freshwater is used to dilute wastewater discharges. The demand for freshwater is expected to rise by 70% by 2050 [1] driving an urgent need to understand the impacts of treated waste effluent discharges on aquatic ecosystems. Wastewater treatment works (WWTW) effluents contain tens of thousands of chemicals, including pharmaceuticals and natural steroid estrogens that are biologically active at low (ng/L) exposure concentrations [2]. However, the long-term consequences of exposure to most of these chemicals on fish health and population sustainability are not known.

There is substantial evidence showing that experimental exposure of fish to WWTW effluents and the estrogens they contain can result in adverse health effects, including effects on reproductive development and breeding output. This has led to concerns that freshwater fish populations might also be affected with cascading consequences for freshwater ecosystems. Feminization of male fish is widespread in stretches of rivers downstream of WWTW outfalls as demonstrated in studies using wild [3,4] and caged [5,6] fish. Feminized phenotypes include the presence of vitellogenin, a female-specific protein in the blood of male fish [7] and the intersex condition: the presence of oocytes and/or female reproductive ducts in otherwise male gonads [3]. Feminization has been attributed to the presence of estrogens in effluents: estradiol (E_2_) and estrone (E_1_) from human excretion; 17 alpha-ethinylestradiol (EE_2_), a component of the female contraceptive pill [8]; and a large number of other estrogenic chemicals from industrial and domestic effluents. WWTW effluents can also induce genotoxic effects [9], alterations in immune function [10], decreased reproductive output [11], altered stress response [12] and changes in reproductive behavior [13].

Concern about estrogens in rivers in the United Kingdom drove a £40 M programme to evaluate the efficacy of various tertiary treatment processes in the removal of estrogens [14,15]. Implementation of such processes will, however, incur considerable costs and a greater carbon footprint for WWTW [14,16], emphasising the need to understand better the population-level consequences for exposure to estrogenic and other so-called endocrine disrupting chemicals (EDCs).

A critical question is whether chronic exposure to estrogenic effluents negatively impacts the viability of wild fish populations, but this has been difficult to address experimentally as it requires controlled experiments extending over periods of several years. Limited studies suggest that high concentrations of EE_2_ (between 3 to 6 ng/L) in the aquatic environment could be a threat to the sustainability of fish populations. For example, a controlled exposure of an entire lake to EE_2_ in Canada resulted in the collapse of the fathead minnow (*Pimephales promelas*) population within three years [17]. Likewise, long-term (>204 days) laboratory exposures of a range of fish species have resulted in the absence of breeding males [18-20] and a three-year exposure of roach (*Rutilus rutilus* L.) to an undiluted WWTW effluent in large tanks resulted in an all-female population [21]. It is not known, however, if this occurs in rivers contaminated by effluents. Female fecundity can also be reduced through estrogen exposure, which can potentially reduce population growth rates [22]. Although the exposure concentrations in these studies were high compared to those typically experienced by wild fish populations [23], exposures to EE_2_ at concentrations below 1 ng/L during the period of sexual development, have been shown to result in feminized gonads in roach [19] and decreased egg fertilization and female-skewed sex ratios in fathead minnows [24]. Evidence from wild roach living in UK rivers has similarly shown that feminized fish (generally less than 10% of males) with large numbers of eggs in their gonads have impaired semen quality [25] and severely (up to 76%) reduced reproductive success [26].

While these studies suggest that exposure to high concentrations of effluent could threaten the viability of fish populations, aggregates of cyprinid fish, including roach, are often found in effluent contaminated rivers. However, numbers alone may provide a misleading assessment of population sustainability as these could be sink populations maintained by substantial immigration from less contaminated locations where successful reproduction still occurs. Likewise, effective population sizes (*N*_e_) – related to the breeding population of fish – may be decreased without necessarily impacting on population sizes [27], as effluent exposure can affect the number of reproducing individuals and can skew reproductive success [21,26]. Density-dependent growth and survival can also play an important role [28], so a few reproducing individuals can potentially maintain large adult population sizes. Indeed, studies in several species of marine fish with high fecundity have shown that *N*_e_ can be several orders of magnitude smaller than census population sizes. One study found two populations of the exploited New Zealand snapper (*Pagrus auratus*) to have values of *N*_e_ less than 1,000 despite adult census population sizes in the millions [29]. Similarly, a study of striped bass (*Morone saxatilis*), a freshwater fish species, found cohorts to consist of a few, full sib families, despite an adult census size of over 300,000 [30]. Critically, *N*_e_ influences long-term sustainability as it determines the rate at which genetic diversity is lost from a population through genetic drift [31]. High genetic diversity increases the long-term potential for populations to adapt to changes in the environment and also acts to reduce the risk of inbreeding [32]. Small *N*_e_, however, may act to increase the chances of losing some lethal or sub-lethal mutations through genetic purging.

Understanding the impact of estrogenic effluents on the sustainability of fish populations is, therefore, paramount, but has been limited to date by the logistical challenges involved in undertaking long-term exposures to realistic effluent concentrations, and understanding the demographic history of wild fish populations at highly contaminated sites. In this study, we examine evidence for population impacts on wild roach (*R. rutilus*), a fish species in which feminization is widespread, in southern England. Southern England has some of the highest proportions of WWTW effluent in rivers known globally, and numerous weirs and locks which potentially confine fishes to heavily polluted stretches of river. We have used this system to evaluate whether stretches of river highly contaminated with estrogenic effluents have impaired breeding populations of roach. To do this we undertook analysis of population genetic structures of roach in the region using DNA microsatellite analysis. Microsatellite data were also used to calculate *N*_e_ and estimate levels of gene flow to determine the extent to which these populations are maintained through immigration of fish from less contaminated stretches of river.

## Results

### Genetic diversity and genetic bottlenecks

**Figure 1:**
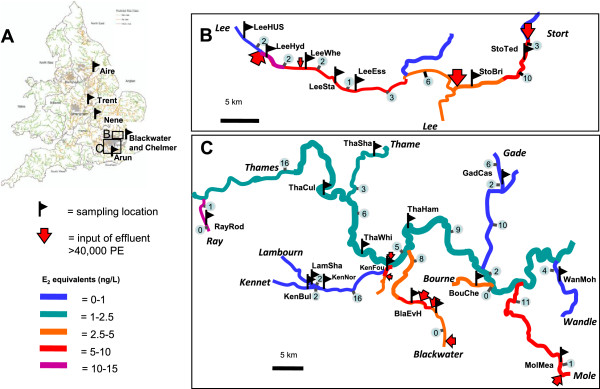
**Locations of sample sites in England. (A)** Modified from Williams *et al*. [33]. For **(B)** and **(C)**, numbers in circles represent the number of obstructions to fish movement (either weirs or locks). Locks in the Kennet and Thames below ThaWhi have fish passes for salmon movements, but are likely to represent a barrier to movement of roach. In the upper river Lee, only weirs over 1 m are shown. PE = population equivalents, which relates to the size of the population served by the waste water treatment works. The different colours used to depict the rivers represent predicted mean estrogenicity (E2 equivalents in ng/L) [34].

A total of 1,769 roach, constituting 39 samples (roach sampled from one location within a river – typically a stretch of approximately 100 m – at one time point) from 32 different geographic locations in England were genotyped (Figure 1). Data for 14 microsatellite loci [see Additional file 1 for details] revealed high genetic diversity in all 39 samples (Table 1). Allelic richness (*AR*) ranged from 6.8 to 8.9, and expected heterozygosity (*H*_e_) ranged from 0.69 to 0.75 (Table 1, see Additional file 2 for diversity statistics for each locus). Nevertheless, significant differences in *AR* (analysis of variance (ANOVA), F_(38,532)_ = 2.1398, *P* = 0.00014) and observed heterozygosity (*H*_o_) (ANOVA, F_(38,532)_ = 1.8677, *P* = 0.0017) among samples were detected. Roach sampled at two relatively unpolluted sites (LamSha and LeeHUS’95) exhibited comparatively low *AR* and LeeHyd’95 exhibited comparatively relatively low *H*_o_ [see Additional file 3]. No significant differences in *H*_e_ were found. Additionally, there was evidence for genetic bottlenecks at two relatively unpolluted sites sampled within the rivers Arun (AruHUS) and Lee (LeeHUS), and at a polluted site in the Lee (LeeWhe) (Table 1).

**Table 1:** **Sampling locations, genetic diversity statistics (allelic richness ( *AR *) and expected heterozygosity (*H***_**e**_**)) for each population sample**

**Sample code**^**a**^	**River**	**Year**	**Lat.**	**Long.**	**E**_**2**_**Eq (ng/L)**^**b**^	**No. fish genotyped**	**Bottleneck (TPM)**^**c**^	***AR***	***H***_**e**_	**Ref.**^**d**^
**Blackwater**										
BlaBlM	Blackwater	2010	51.78529	0.651013	4.1	55	0.40	7.9	0.71	
BlaSti	Blackwater	2010	51.88792	0.606184	7.1	55	0.60	8.2	0.72	
CheAbB	Chelmer	2010	51.84596	0.423716	1.2	50	0.06	7.7	0.75	
**Nene**										
NenBro’95	Nene	1995	52.24627	-0.77987	1.2	47	0.69	8.3	0.72	[3]
NenBro’99	Nene	1999	”	”	1.2	47	0.80	8.3	0.73	[25]
NenEct	Nene	2007	52.24234	-0.80281	4.2	51	0.79	8.6	0.73	
**Aire**										
AirDar	Aire	2011	53.79336	-1.54911	2.7	43	0.73	8.6	0.75	
**Arun**										
AruHor’95	Arun	1995	51.05516	-0.36197	4.1	54	0.90	7.8	0.73	
AruHor’00	Arun	2000	51.0556	-0.36124	4.1	34	0.29	7.6	0.73	[25]
AruHor’08	Arun	2008	“	“	4.1	69	0.73	7.9	0.74	[26]
AruHUS	Arun	1995	51.05953	-0.35326	0.2	48	**0.01**	7.4	0.73	[3]
**Thames catchment**										
BlaEvH’10	Blackwater	2010	^e^		4.2	41	0.98	8.5	0.72	
BlaEvH’00	Blackwater	2000	51.327864	-0.769635	8.8	47	0.98	8.3	0.71	[25]
BouChe’11	Bourne	2011	51.38086	-0.47711	4.8	56	0.62	8.3	0.74	
BouChe’02	Bourne	2002	51.40286	-0.54223	5.8	31	0.55	8.8	0.75	
BouChe’06	Bourne	2006	51.40330	-0.54150	5.8	48	0.25	8.3	0.75	[26]
GadCas	Gade	2010	51.65893	-0.42559	NM ^f^	56	0.38	8.1	0.74	
KenBul	Kennet	2010	51.39707	-1.28485	0.6	51	0.82	8.4	0.74	
KenFou	Kennet	2010	51.43564	-0.97664	8.1	32	0.64	8.7	0.75	
KenNor	Kennet	2010	51.40165	-1.33725	0.2	52	0.62	8.5	0.73	
LamSha	Lambourn	2011	51.40816	-1.30843	0.03	41	0.10	6.8	0.72	
LeeEss	Lee	2010	51.77279	-0.18818	6.6	56	0.60	8.3	0.73	
LeeHyd	Lee	2010	51.83958	-0.35825	10.3	28	0.36	8.2	0.73	
LeeHyd	Lee	1995	51.84751	-0.37111	11.6	44	0.62	7.7	0.70	[3]
LeeHUS	Lee	1995	51.84935	-0.37395	NM	37	**0.001**	7.0	0.73	[3]
LeeSta	Lee	2010	51.7894	-0.22496	6.6	31	0.85	8.4	0.71	
Lee’00	Lee	2000	^g^		NM	41	0.90	8.1	0.70	[25]
LeeWhe	Lee	2010	51.81424	-0.28903	6.6	55	**0.05**	8.5	0.75	
MolMea	Mole	2010	51.19028	-0.18581	5.8	42	0.84	8.4	0.73	
RayRod	Ray	2003	51.57093	1.81815	10.9	30	0.40	7.7	0.72	
StoBri	Stort	2010	51.77989	0.05024	4.1	52	0.92	8.1	0.71	
StoTed	Stort	2010	51.83115	0.16892	6.0	30	0.97	8.2	0.69	
ThaCul	Thames	2010	51.65046	-1.26739	1.6	44	0.45	8.8	0.74	
ThaHam	Thames	2010	51.55989	-0.87347	1.8	44	0.45	8.2	0.74	
ThaWhi	Thames	2010	51.48662	1.08974	1.5	60	0.88	8.2	0.74	
ThaSha	Thame	2010	51.75281	-1.03319	1.9	50	0.38	7.9	0.74	
WanMoh	Wandle	2011	51.40329	0.18821	3.3	48	0.33	8.2	0.75	
**Trent**										
TreWol	Trent	1995	52.781178	-1.971789	3.7	45	0.55	8.0	0.72	
TreNot	Trent	2007	^*g*^			24	0.50	8.9	0.75	

Full diversity statistics for each locus are given in Additional file 8 and statistical differences in genetic diversity are given in Additional file 3. ^a^Headings in bold indicate different catchments; ^b^Estradiol equivalents, the predicted average estrogenicity at the sample site, calculated from the predicted concentrations of E_1_, E_2_ and EE_2;_^c^TPM = two phase model for microsatellite evolution used for this test; ^d^Source of samples; ^e^A composite sample of fish caught at BlaEve (51.354119, -0.8584853) and BlaHaw (51.324119, -0.7665606). Effluent concentrations are an average between the two sites for statistical analysis;

^f^NM = not modelled. For this site there are no major upstream discharges. For statistical analysis, and average E_2_Eq value for sites with no major discharges was used. ^g^The exact locations for these sample sites are unknown. Numbers in bold indicate significance (p ≤ 0.05).

### Population genetic structure of roach in English rivers

**Figure 2:**
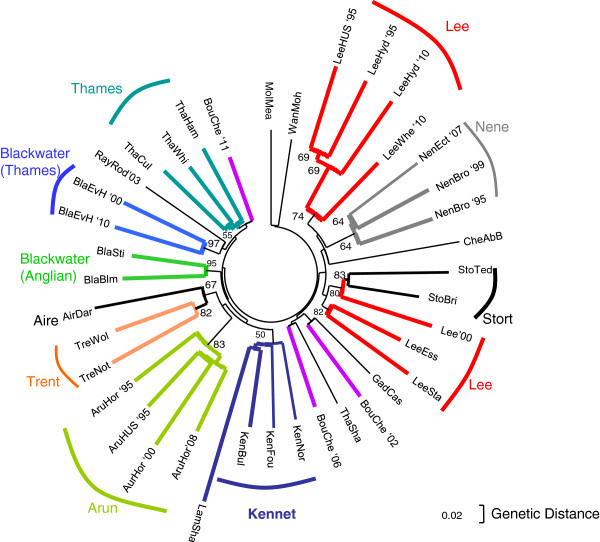
**Neighbour-joining phylogenetic tree for the 39 roach population samples.** The tree is based on the data from 14 microsatellite loci using chord distance from Cavalli-Sforza and Edwards [36]. Only bootstrap values above 50% are shown. Numbers at the end of sample codes indicate years that populations were sampled.

We undertook an analysis of population genetic structure in order to examine the genetic similarity of roach between and within catchments. Analysis of molecular variance (AMOVA) indicated the majority of variation was partitioned among individuals within river locations, with river location accounting for a small 2.27%, but highly significant proportion of the genetic variation (Table 2). Average pairwise *F*_ST_ between roach samples from different catchments was 0.028 and comparisons were consistently highly significant (Table 3, Additional file 4). The population tree (Figure 2) shows distinct clusters of samples in different catchments: the Arun, the Nene, the Anglian Blackwater and the Trent, supported with moderate–high bootstrap values (>64%). Samples from the Arun and the Nene also group in the principal component (PCA) and the STRUCTURE analyses [see Additional file 5 and Additional file 6] demonstrating a distinct genetic identity of fish at these sites. Utilising the method of Evanno *et al.*[35], the inferred most likely number of genetically distinct clusters in the STRUCTURE analysis was three, comprising: the Arun, the two most upstream sample sites in the river Lee, and all remaining sites genotyped. However, from visual examination of STRUCTURE plots run with higher levels of *K* [see Additional file 6] other possible groups are apparent. We found no evidence that roach in the Thames catchment constitute a distinct genetic group, as samples failed to group together in any analysis (Figure 2, Additional file 5 and Additional file 6). This may reflect a true lack of genetic distinctiveness of roach in this catchment, but may also result from the limited ability of the microsatellite markers used to resolve population genetic structure at this level.

**Table 2:** Analysis of molecular variance (AMOVA) testing for partitioning of genetic variation among roach samples, grouped according to geography

**Source of variation**	**d.f.**	**Sum of squares**	**Variance**	**% Total**	***P*-value**
**Geographical partition, location**^**a**^					
Among locations	32	584.063	0.11631	2.27	<0.00001
Among samples within locations	6	35.197	0.00953	0.19	0.03226
Within samples	3,499	17,489.296	4.99837	97.54	<0.00001
Total	3,537	18,108.556	5.12421		
**Geographical partition, catchment**^**b**^					
Among groups	5	189.529	0.06309	1.22	<0.00001
Among samples within groups	33	429.731	0.08909	1.73	<0.00001
Within samples	3,499	17,489.296	4.99837	97.05	<0.00001
Total	3,537	18,108.556	5.15055		

^a^Samples from same location caught in different years are grouped; ^b^Samples grouped by catchment (Aire, Arun, ‘Blackwater and Chelmer’, Nene, Thames and Trent).

**Table 3:** Summary of pairwise ***F***_**ST **_**and *D***_**est**_ among roach samples (see Additional file 4 for full table of values)

	***F***_**ST**_	***D***_**est**_	***P*-value**^**a**^
**Between catchments**			
Thames/Arun	0.033, (0.002-0.074)^b^	0.0617, (0.0124-0.1612)^b^	
Thames/Nene	0.026, (0.007-0.076)	0.0453, (0.0126-0.1458)	
Thames/Trent	0.026, (0.008-0.069)	0.0447, (0.0059-0.1394)	
Thames/Chelmer, Blackwater	0.022, (0.005-0.057)	0.0444, (0.0035-0.1191)	
**Within catchments**			
Thames	0.022, (-0.004-0.090)	0.0376, (-0.0058-0.1914)	
Arun	0.007, (0.002-0.014)	0.0064, (0.0015-0.0104)	
Nene	0.002, (0.000-0.003)	0.0010, (-0.0001-0.0020)	
Blackwater/Chelmer	0.022, (0.005-0.057)	0.0454, (0.0058-0.0652)	
**Neighbouring samples in the river Lee/Stort**			
LeeHUS/LeeHyd’95	-0.002	0.0017	0.78
LeeHyd’10/LeeWhe	0.020	0.0352	>0.00001
LeeWhe/LeeSta	0.009	0.0189	>0.00001
LeeSta/LeeEss	0.009	0.0127	>0.00001
LeeEss/StoBri	0.015	0.0349	>0.00001
**Other neighbouring stretches**			
BlaBlM/BlaSti	0.007	0.0058	>0.00001
AruHUS/AruHor’95	0.002	0.0043	0.22

^a^For *F*_ST_ estimate; ^b^average, (range). Pairwise values for neighbouring stretches in the Lee/Stort, the Anglian Blackwater and the Arun are also given.

### Population genetic structure of roach within rivers and catchments

Despite Thames catchment roach appearing not to constitute a single genetic unit, distinct from roach in other regions, the study did find evidence for significant genetic structuring in roach populations within the Thames catchment. This suggests the existence of local subpopulations exchanging a limited number of effective migrants (breeding individuals) rather than panmixia (where all individuals are potential partners). For example, average *F*_ST_ between samples in the Thames catchment was 0.022, only slightly lower than the average for between-catchment comparisons (0.028) for the study as a whole, while 262 of the 325 pairwise *F*_ST_ comparisons in the catchment were highly significant. There was a weak, but significant, relationship between genetic and geographic distance (r^2^ = 0.1089, *P* = 0.010) within the catchment, indicating a tendency for individuals to produce offspring with fish from nearby populations rather than distant populations [see Additional file 7]. Additionally, the population tree (Figure 2) and PCA analyses [see Additional file 5] showed groups comprising samples from neighbouring Thames sites: three from the main Thames; four from the Kennet and its tributary (Lambourn); samples from the Stort and the Lee; and samples from the Wandle and Mole. Samples from the Thames Blackwater collected in the years 2000 and 2010 (approximately two to three generations) clustered with very high bootstrap support (98%) in the population tree (Figure 2). This indicates that this roach population is largely restricted to this stretch of river, which includes both moderately and highly polluted sites, and has no substantial uncontaminated upstream stretch (Figure 1).

Despite the proximity of some populations in the PCA and tree, we also found significant genetic differentiation between samples from some neighbouring stretches of the same river, sometimes occurring over small distances of separation (<10 km), for example, within the upper Lee (see below), between the Lee and the Stort, between the Blackwater and main Thames, between the Lambourn and the Kennet and within the Anglian Blackwater. In other cases, despite the separation of sampling locations by in-river impoundments such as weirs, we found no significant genetic differentiation between sites, for example, within the Stort, the main Thames, the Kennet, the Arun, the Nene and the Trent. Thus, patterns of within-river genetic structure differed between river stretches. For some other fish species analysis of genome-wide SNP data has provided greater resolution in population structure than that achievable using microsatellite data [37], and it is possible that some fine-scale genetic structure in the roach populations has not been detected with the microsatellites used in the current study.

### Relationship between exposure to estrogenic effluents and effective population size

**Figure 3:**
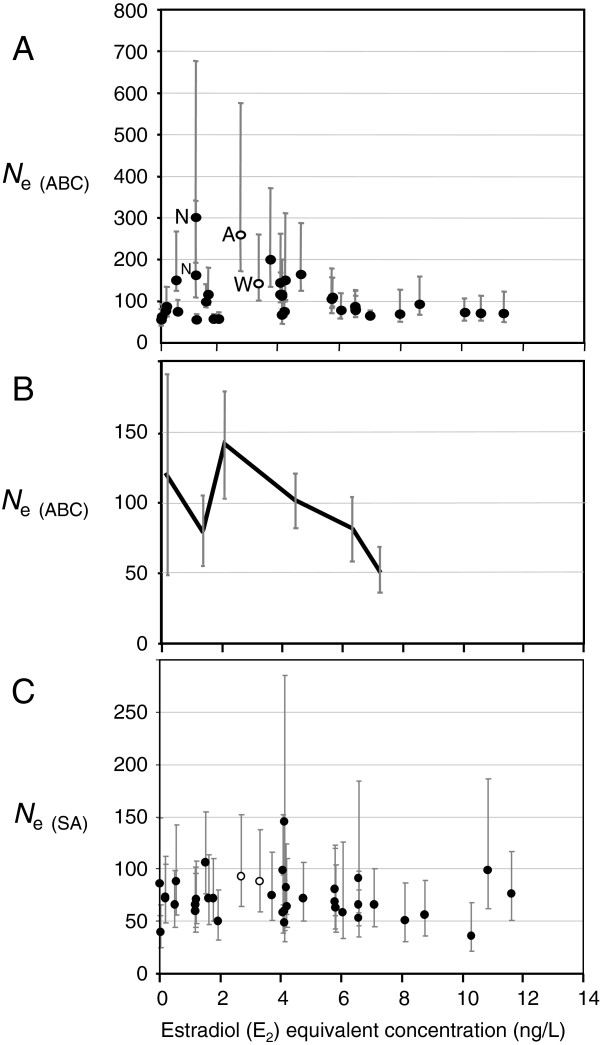
**Effective population size (*N***_**e**_**) plotted against predicted estrogen exposure for 37 population samples of *Rutilus rutilus*. (A)***N*_*e*_ calculated using the Approximate Bayesian Computation (ABC) method in the program OneSAMP [38]. Tests for homogeneity of variances: Bartlett’s, *P* = 0.0036, Levene’s, *P* = 0.17. **(B)** Results of binning analysis for data shown in A; each bin which encompasses all data points starting at the lower point represented by the mean and standard error up to, but not including, the next bin. **(C)***N*_e_ calculated using the sibling assignment (SA) method in Colony [39]. In A and C, error bars are 95% confidence intervals. In cases in which more than one population had similar values, data points overlie each other; thus, individual data points are not always visible. These plots include estimates from sample sites sampled in different years, for example, in the River Nene (N) (which were averaged for statistical analysis) and sites where recent restocking had occurred (open circles: River Aire (A), River Wandle (W)), which were excluded from the statistical analyses.

Estimates of *N*_e_ calculated from the microsatellite data using the approximate Bayesian computation method (*N*_e(ABC)_), ranged from 54 to 301 for each sample, with higher precision for small *N*_e_ estimates (Figure 3A). We found no evidence for a correlation between *N*_e(ABC)_ and predicted E_2_ equivalents (E_2_Eq), a measure of total estrogenicity of the river water due to contamination by sewage effluent (generalized linear models (GLM), F_(1,20)_ = 0.7468, *P* = 0.40) or for an interaction between sample site and estrogen exposure (GLM, F_(6,19)_ = 1.9954, *P* = 0.14) across the 28 sample sites where no recent restocking had occurred and had sufficient sample sizes for robust *N*_e_ calculation. However, the 95% confidence intervals (CI) for the model coefficient indicated *N*_e(ABC)_ could decrease by a maximum of 5.6% for each incremental increase in exposure of 1 ng/L E_2_Eq, or 65% at 11.6 ng/L E_2_Eq, equivalent to the most polluted river stretch included in this study. The inclusion of roach density as an additional covariate within the model also produced a non-significant result (GLM, F_(1,16)_ = 1.3966, *P* = 0.26), albeit for a reduced number of sites (19). Similarly, there was no significant correlation between the other variables included in the statistical analyses (average flow rate, geographic/phylogenetic group and roach density) with *N*_e(ABC)_. This analysis makes the assumption that immigration of fish from remote sites is limited and this is discussed below.

There was limited evidence for reduced variation in *N*_e_ in roach sampled from more contaminated stretches of river compared to those sampled from less contaminated sites; all estimates above 6 ng/L E_2_Eq were below 100, whereas there was greater variation (54 to 301) where E2Eq was below 6 ng/L (Figure 3B). Estimates of *N*_e_ using other methods were of the same order of magnitude but had wider confidence intervals for each estimate; *N*_e(SA)_ ranged from 36 to 145 and also showed no relationship with E_2_Eq (Figure 3C). Temporal estimates of *N*_e_, calculated from allele frequency changes over several generations using the Jorde and Ryman method [40], varied from 14 to 265, but were available for too few locations to make meaningful comparisons (Table 4). Overall, the relatively small variations in *N*_e_ observed in this study could not be explained by any of the environmental or other variables measured in this study.

**Table 4:** **Temporal estimates of effective population size (*N***_**e**_**) among roach samples**

**Site**	**Time interval**	**G**^**a**^	***N***_**e **_**(95% CI)**^**b**^	***N***_**e **_**(95% CI)**^**c**^
Nene	1995 to 1999	1	265 (66-∞)	619 (82-∞)
AruHor	1995 to 2000	1	14 (8–79)	48 (26–137)
	2000 to 2008	2	60 (31–1733)	321 (106-∞)
	1995 to 2008	3	73 (43–247)	232 (123–669)
BouChe	2002 to 2006	1	32 (17–219)	51 (25–151)
	2006 to 2011	1	45 (23–9572)	63 (35–162)
	2002 to 2011	2	87 (44–3897)	495 (295–1405)
LeeHyd	1995 to 2010	3	137 (81-∞)	346 (145–35518)
BlaEvH	2000 to 2010	2	141 (68-∞)	206 (83-∞)

^a^G = assumed number of generations between sampling points; ^b^Calculated using the Jorde and Ryman method [40]; ^c^Calculated using the classical moment-based method of Waples [41]. CI, confidence interval.

### Population genetic structure within the River Lee, a high effluent river

**Figure 4:**
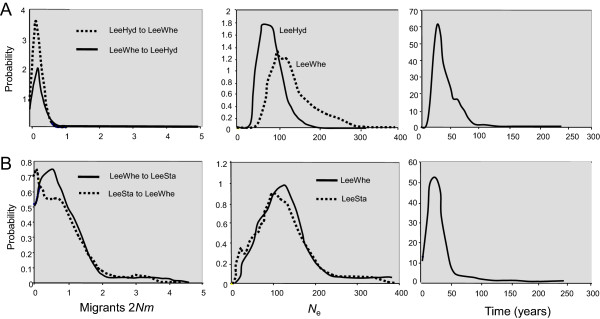
**Posterior probabilities of migration rates, effective population sizes (*N***_**e**_**) and time since divergence estimated using IM**_**A**_**2 [**[42]**] for two pairs of roach populations in the River Lee. (A)** Estimates for parameters calculated for the LeeHyd’10 and LeeWhe and **(B)** for LeeSta and LeeWhe. These *N*_e_ values are influenced by average *N*_e_ since the initial split of the populations.

The average proportion of effluent in the upper Lee downstream from Harpenden and East Hyde WWTWs ranges from 28% to 70% in different stretches. Significant genetic differentiation was detected between fish sampled from four of the five locations in this stretch of river (*F*_ST_ values ≥0.009), and between these samples and two from its tributary, the Stort (*F*_ST_ ≥0.015) shown in Table 3. The presence of numerous large weirs (Figure 1) likely confines fish to particular areas of this river; nonetheless, samples from the Lee and the Stort did cluster together in some analyses (Figure 2, Additional file 5). A sample from the upstream, unpolluted sample site (LeeHUS) grouped with two samples (collected in 1995 and 2010) from the most polluted river stretch immediately downstream (LeeHyd). The next sample site downstream, LeeWhe, was distinct from these (Figure 2, Additional file 5 and Additional file 6), indicating restricted movement of fish between LeeHyd and LeeWhe over at least three to five generations (Figure 2). Analysis using the program IM_A_2 [42] suggested that there was less than one effective (breeding) migrant per generation between LeeHyd and LeeWhe in either direction, and about one migrant per generation from LeeWhe downstream to LeeSta (Figure 4). Collectively, these data suggest that roach populations at LeeWhe and those downstream do not rely on migration from the uncontaminated stretch of this river. Despite this, *N*_e(ABC)_ estimates for these polluted sites in the upper Lee ranged from 70 to 84 (95% CI: 50 to 127) compared to only 54 (95% CI: 42 to 82) for the upstream uncontaminated location, suggesting no substantial impact of the effluent on the effective population size of these roach.

## Discussion

In this study, analyses of population genetic structure *via* the analysis of DNA microsatellite loci identified distinct subpopulations of roach in two tributaries of the Thames, the rivers Lee and the Blackwater, that were largely restricted to high-effluent stretches of the rivers over multiple generations. This is despite evidence for widespread feminization of male fish in the studied rivers and previous evidence that feminization alters breeding capabilities [21,26]. Both of these tributaries contain feminized fish [25]), with predicted average exposure of between 4 and 9 ng/L E_2_Eq. We also found no statistically robust evidence for a substantial impact of estrogenic sewage effluents on *N*_e_ of roach. The possibility of a reduction in *N*_e_ of up to 65% for roach living in the most polluted river stretches (E_2_Eq of 11.6 ng/L) could not be ruled out, due to the wide 95% confidence intervals associated with the statistical model. Moreover, our analysis included relatively few samples from rivers in the highest risk category, largely because these sites are rare.

### Caveats

As with any modelling exercise, this analysis makes assumptions that may affect the interpretation of the results. One of these assumptions is that migration between sites with different pollution profiles is limited over two to three generations, the time frame likely to have the greatest influence on *N*_e(ABC)_[43]. This was ensured by selecting sites with physical obstructions between them. However, quantifying migration rates over this timescale was not always possible because all potential source populations could not be sampled and, in some cases, we found no significant genetic differentiation between roach at sites distant from one another. Genetic differentiation can take many generations to manifest with low levels of migration [44]. Histological data from the Arun and the Lee show that feminized gonads in roach were approximately 6-fold (Lee) and approximately 2.5-fold (Arun) more prevalent in populations living in the stretches downstream of major WWTW inputs compared with those living upstream [3]. This demonstrates that migration in these rivers was indeed restricted to stretches delimited by physical barriers, despite no significant genetic differentiation observed between river stretches (*F*_ST_ <0.002, Table 3).

A second assumption is that no restocking of the rivers sampled had occurred or that the effect of restocking activities on *N*_e(ABC)_ was relatively minor. Approximately 500,000 hatchery-reared roach just over one-year-old (so called ‘1+’ fish) have been introduced into the Thames catchment since 2000; broodstock for hatchery fish originate from the river Trent. The influence of restocking activities on *N*_e(ABC)_, however, is likely to be relatively minor, as the sites sampled in this study were separated by major physical barriers from sites where these introductions had occurred. Moreover, introductions prior to 2000 are unlikely to have had a large influence on *N*_e(ABC)_ as this is primarily affected by the size and variance in reproductive success of the parental generation, which would have spawned between 2004 and 2007 for most of the samples in this study. However, we cannot exclude some influence of introductions prior to 2000 as some of the summary statistics used to calculate *N*_e(ABC)_ are known to be affected by demographic processes over a longer time period [38]. The effects of introductions on genetic diversity, the detection of bottlenecks and population structure are likely to be greater, as these factors are affected by demography over many generations. However, neither the success of the reintroduced fish nor the size of the roach population in the Thames is currently known. In salmonids, restocking success is highly variable and has been attributed to local adaptation [45]. Using our microsatellite dataset, 73% of 48 individual roach from a stretch of the Wandle (restocked in 2007, 2009, 2010) assigned to the Thames reporting regions. Only 5% (two fish) assigned to the Trent (the source of the parents of introduced fish), which may be mis-assignments, as 5% also assigned to the Arun, 10% to the Anglian Blackwater and 10% to the Nene, from where no restocking had taken place. Thus, the success of the re-introduced fish may be low, but this requires further investigation.

### Evidence for self-sustaining populations in effluent contaminated rivers

While this study does not exclude the possibility that estrogenic effluents reduce *N*_e_ of fish populations, it suggests that roach populations can be self-sustaining despite exposure to estrogens over several generations. These findings are consistent with the fact that the prevalence of male fish with moderate to severely feminized gonads (that have been shown to have substantially reduced reproductive competitiveness in controlled breeding studies) is generally less than 10% in English rivers [3,25,26,46]. The reproductive competitiveness of fish with the more common mild-intersex condition is similar to those of fish without gonadal feminization [26]. In roach, the gonads of male fish exposed to estrogens become progressively feminized with age [46], so gonadal feminization could theoretically increase *N*_e_ by reducing the reproductive dominance of large older males in estrogen-contaminated rivers. While the effects on females are less well studied, female roach exposed to an undiluted effluent for three years in large tanks were able to breed, despite the fact that this exposure caused complete gonadal feminization of males [21]; similarly, the majority of females collected from two effluent-polluted rivers examined in this study were also able to breed [26].

### Population risks of long-term exposures to estrogenic effluents

The results of this study on wild roach populations seem to contrast with studies that have assessed population risk through long-term exposures to estrogens, where exposure to concentrations between 3 and 6 ng EE_2_/L [17-20] or to a full-strength effluent [21] resulted in all-female populations and/or reproductive failure. The apparent difference between the wild populations and those experiments designed to simulate ‘real world’ exposure, however, may be because the fish living in the effluent-contaminated rivers examined in this study have been exposed to a lower level of estrogen or because all of the estrogen is not bioavailable; organic pollutants can bind to particulates and dissolved organic matter [47]. The most contaminated river in this study has a mean proportion of effluent of approximately 70%, although the majority of contaminated English rivers average approximately 10% to 30% [34]. While EE_2_ has been measured up to approximately 4 to 8 ng/L in English WWTW effluents [33,48], for the most part, they are lower [23,49], and estrogen concentrations vary greatly over short periods. For instance, EE_2_ was detected in only 21 of 135 water samples from the Lee, although occasionally reaching 4 ng/L [33]. Considering the totality of estrogen content, the predicted average estrogenicity of the most contaminated site in this study is 12 ng/L E_2_Eq and would be below 21 ng/L for 90% of the time. Only 1% to 3% of 10,313 individual river reaches in the UK receiving WWTW effluent were predicted to have average E_2_Eq >10 ng/L, and, of these, many are ditches composed almost entirely of sewage effluent [34]. As E_2_ is approximately 10 times less potent than EE_2_ in inducing gonadal feminization in fish [34,50], it is probable that average life-time exposure to estrogens in the wild does not currently reach the concentrations shown to cause sex-reversal and population collapse in controlled experimental exposures. Green *et al*. [51] recently predicted a doubling of estrogen exposure concentrations in some rivers with population growth and climate change by 2050 suggesting an increased likelihood of population level effects of estrogenic effluents in the future, unless mitigated by substantial improvements in sewage treatment processes.

### Influences on the population genetic structure of roach

The population genetic structure of roach in southern England observed in this study may have been influenced by historical biogeography, migratory behaviour, human translocations and in-river barriers. Roach can be highly mobile and can migrate over 10 km, particularly in the spawning period April to June, if migration is not obstructed [52]; additionally, there is some evidence that roach show fidelity in migration and return to spawning sites they have used previously [53]. Within the Thames catchment, the observed population genetic structure likely results, at least in part, from the large number of physical barriers, such as weirs and locks (Figure 1); these have been recognized as major factors restricting movement (including downstream) of roach [54]. Similarly, the importance of barriers in driving intra-catchment genetic variation is well documented in other fish species, for example, brown trout [55]. Only obstructions in the main River Thames and the Kennet are equipped with fish passes and, although some passes can be used by roach [56], the effectiveness of these passes in allowing fish movement has not been studied. As we identified significant genetic differentiation between roach from the Kennet and the Thames, despite being connected by fish passes, these passes may represent major physical separation barriers to this fish species.

## Conclusions

Despite the widespread feminization of male roach in effluent-contaminated rivers of southern England, using nuclear DNA microsatellites we were able to identify some populations that have been confined to stretches of river with moderate to high exposure to estrogenic effluents over multiple generations. We also found no evidence of a correlation between the *N*_e_ of roach populations and predicted exposure to estrogens, although because of the wide confidence intervals, a reduction in *N*_e_ of up to 65% is still possible at the most contaminated sites.

## Methods

### Study location

Southern England, particularly the region within the Thames catchment, was chosen for this study for four reasons. Firstly, it is a densely populated region with relatively low rainfall and, therefore, includes some river stretches with some of the highest concentrations of WWTW effluents in the United Kingdom [34]. Secondly, feminization of roach has been widely reported in the region [3,46]. Thirdly, many rivers in the region have locks, dams or weirs which are likely to limit movement of fish species between stretches of river with different pollution profiles. Fourthly, the effluent concentrations and risk of estrogenic endocrine disruption have been modelled [34]. Sample sites are shown in Figure 1 and Table 1 and were selected to span the full range of predicted estrogen concentrations in English rivers and where obstructions are likely to restrict fish movements [3,46].

### Roach study species

Roach was selected as the study species because it is native and widely distributed in the United Kingdom, including in rivers polluted with WWTW effluents. Additionally, widespread feminization has been reported in wild populations and with a proven association with exposure to estrogenic effluents [14,19,21,57]. Roach generally reach sexual maturity between two and three years and spawn annually in the spring. Adult roach can migrate considerable distances, but where weirs obstruct upstream and downstream movement they are able to complete their lifecycles in a single stretch of river [54].

### Population-genetic analyses

To understand the extent to which roach populations are restricted to various stretches of river, several approaches were used to investigate population genetic structure. We analysed microsatellite loci variation in 1,769 fish sampled between 1995 and 2011. Each fish was genotyped at between 14 to 19 microsatellite loci. Microsatellite genotypes are provided in Additional file 8. Protocols for DNA extraction and details of amplification of the microsatellite loci are illustrated in Additional file 1. Data for 14 microsatellite loci were used to calculate three measures of genetic diversity: observed heterozygosity (*H*_O_) and expected heterozygosity (*H*_e_) using GenAlEx 6 [58]; allelic richness (*AR*) was calculated using Fstat v2.9.3 [59] – see Additional file 1 for full details. The programme BOTTLENECK [60,61] was used to test for recent genetic bottlenecks. This programme tests for a relative excess in heterozygosity that is apparent for a few generations after a bottleneck and develops because allelic diversity declines faster than heterozygosity, due to loss of rare alleles. Pairwise genetic differentiation between the sampled sites was estimated using *F*_ST,_ calculated using Arlequin 3.5 [62] and Jost’s *D*, *D*_est_[63], calculated using SMOGD [64]. The significance of the *F*_ST_ estimates was assessed based on 10,000 permutations. AMOVA was performed using Arlequin. In order to test whether fish are more likely to produce offspring with local mates, compared to mates in geographically distant locations within the Thames catchment, isolation by distance analysis was performed using the Mantel test [65] in GenAlEx 6 [58]. Genetic similarity between populations was investigated using population based trees, calculated in POPULATIONS, v1.2.30beta [66], PCA in GenAlEx 6 [58] and a Bayesian clustering approach in STRUCTURE [67]. Finally, the program IM_A_2 [42,68] was used to estimate migration rates between adjacent populations within high effluent stretches of the Lee, giving relatively high pairwise *F*_ST_ values (LeeHyd, LeeWhe, and LeeSta). See Additional file 1 for further details. To investigate the influence of restocking, genetic assignment of fish from the Wandle was undertaken using the ‘leave one out test’ in the computer program, ONCOR [69], based on their microsatellite genotypes. The reporting regions comprised: (1) the Wandle, (2) Lee/Stort, (3) rest of the Thames, (4) Trent, (5) Nene, (6) Arun, (7) Chelmer and (8) Anglian Blackwater. All animals used in this research were treated humanely and with regard for the alleviation of suffering; all procedures were subject to approval by the local ethical review process as required under the U.K. Animals (Scientific Procedures) Act (1986).

### Effective population size

To test whether WWTW effluents substantially reduce the size of breeding populations, effective population sizes (*N*_e_), which relate to the number of breeding fish and skews in breeding success, were estimated using the microsatellite genotypes. We compared *N*_e_ from sites ranging from little/no upstream WWTW effluent inputs to those where the majority of the flow can comprise WWTW effluent. Two single sample (generation) methods, that use different aspects of the microsatellite data, were used to estimate *N*_e_ for each population; the Approximate Bayesian Computation (ABC) method using ONeSAMP 1.2 [38], hereafter referred to as *N*_e(ABC)_; and the sibling assignment method (SA), *N*_e(SA)_[39]. Temporal estimates for *N*_e,_ which are calculated from the change in allele frequencies between generations, were also estimated for sites where fish had been sampled more than once using TempoFs [40] and NeEstimator [70]. For further details see Additional file 1.

### Statistical analysis

GLM were used to examine the relationship between predicted exposure to estrogenic effluents and *N*_e(ABC)_. For further details see Additional file 1. Differences in genetic diversity among sampled populations were tested using ANOVA. All statistical analysis was performed using the software R 2.13.0 [71].

## Abbreviations

AMOVA: analysis of molecular variance; ANOVA: analysis of variance; AR: allelic richness; CI: confidence interval; E1: Estrone; E2: Estradiol; EE2: 17 alpha-ethinylestradiol; GLM: Generalized Linear Models; He: expected heterozygosity; HWE: Hardy-Weinberg equilibrium; MCMC: Markov chain Monte Carlo; MEGA: Molecular Evolutionary Genetic Analysis; Nb: effective number of breeders; Ne: effective population size; Ne(SA): effective population size estimate calculated using the sibling assignment method; Ne(ABC): effective population size estimate calculated using the approximate Bayesian computation method; PCA: principal component analysis; SNP: single nucleotide polymorphism; WWTW: Waste Water Treatment Work (WWTW).

## Competing interests

The authors declare that they have no competing interests.

## Authors’ contributions

PH, EN, JS, SJ, JS, JS and CT participated in the design of the research. RW modeled estrogen exposure in rivers. PH, EB and EN participated in data collection, analysis of microsatellite data and statistical analysis. PH, EN, EB, RW, JS, SJ, JS and CT wrote the paper. All authors read and approved the final manuscript.

## Authors’ information

PH, the corresponding author, is a molecular ecologist and evolutionary biologist, and is a Research Fellow at the Biosciences Department at the University of Exeter.

## Supplementary Material

Additional file 1 Details of microsatellite genotyping methods, population-genetic analysis and statistical analysis. Click here for file

Additional file 2 Genetic diversity estimates for each locus in each population. Click here for file

Additional file 3 **Graph showing statistical differences in genetic diversity among roach *Rutilus rutilus* populations.** Click here for file

Additional file 4 **Pairwise *F***_**ST**_** and *D***_**est**_** among roach population samples.** Click here for file

Additional file 5 **Multidimensional scaling plots of pairwise *D***_**A**_** distances [**[72]**].** Click here for file

Additional file 6 **Structure analyses plots using the *locprior* model and analysis of optimum number of genetic units in Structure Harvester [**[73]**].** Click here for file

Additional file 7 Correlation between genetic distance and geographic distance (km) between pairs of sites for 24 population samples from the Thames catchment. Click here for file

Additional file 8 Microsatellite genotypes for 1,769 roach genotyped in this study. Click here for file
