# Supplementary material for: Populations of a cyprinid fish are self-sustaining despite widespread feminization of males
Source: BMC Biol. 2014 Jan 13;12:1. doi: 10.1186/1741-7007-12-1 (PMC3922797; doi:10.1186/1741-7007-12-1)
Supplement: Additional file 1 — Details of microsatellite genotyping methods, population-genetic analysis and statistical analysis. [file 1741-7007-12-1-S1.doc]

**Additional file 1 – Detailed Methods and Primer Sequences**

***Choice of sample sites***

Sites were chosen where sufficient numbers of roach could be obtained for robust calculation of genetic parameters (see below) and where no recorded introductions had occurred since 2000, based on information obtained for each of these sites from the England and Wales Environment Agency Live fish Movement Database (LFMD). We also aimed to sample from river sites representing the full span of very low and high effluent (and estrogen) concentrations, using the geographical information systems-based model (LF2000-WQX) which predicts the estradiol equivalents (E_2_Eq) using data from each waste water treatment work (WWTW) within a hydrometric area, the domestic population served, the dry weather flow and the type of treatment [1]. The predicted E_2_Eq have been shown to be in good agreement with the measured values [2] and correlate with the actual incidence and severity of intersex in fish found downstream of the WWTWs [3].

***Sample collection***

The biological material (fin clips and scales) for genetic analysis of the roach populations was derived from a combination of archived material from study sites where feminised fish have been previously reported [4-6] and freshly collected material from fish captured *via* electrofishing (2010-2011). Sampling locations are shown in Table 1 and Fig. 1. Two of the river stretches included in this study (LeeHyd and RayRod) are rated as high risk with predicted E_2_Eq above 10 ng/L and are within the top 3% of river locations for estrogen content in the Thames catchment, whereas another 8 are designated as ‘at risk’ but would reach ‘high risk’ 10% of the time, at times of low water flow and poor dilution of effluent [1]. All fish were aged, by counting scale annuli.

***DNA extraction***

DNA was extracted from scale/fin tissue from adult fish using the Chelex [7] or HotSHOT [8] extraction methods.

***Hybrid testing***

Two DNA-based tests were conducted to ensure that fish were not hybrids of roach with either rudd or bream. The first uses allele-specific PCR amplification [9]. The second uses length differences in the internal transcribed spacer (ITS) region between species [10].

***Microsatellite genotyping***

Microsatellite loci were amplified in three PCR multiplexes using the Type-it Microsatellite PCR Kit (Qiagen) with a total volume of 9 μl. The following touchdown PCR cycle for was used for multiplex reaction 1a, 1c and 2: 95 ^o^C for 5 minutes followed by 37 cycles of 95 ^o^C for 30s, annealing temperature for 90s and 72 ^o^C for 3 minutes. Annealing temperatures were 3 cycles at 62 ^o^C, 4 at 58 ^o^C, 5 at 55 ^o^C, 10 at 53 ^o^C, 5 at 51^o^C, 5 at 49 ^o^C and 5 at 47 ^o^C followed by a final extension of 72 ^o^C for 10 minutes and 60 ^o^C for 35 minutes. The following cycle was used for 1b: 95 ^o^C for 90 s, followed by 35 cycles of 30 s at 95 ^o^ C, 45 s at 55 ^o^C and 60 s at 72 ^o^C and a final extension period of 72 ^o^C for 10 minutes and 60 ^o^C for 35 minutes. Amplicons were run on two lanes on a Beckman Coulter DNA sequencer. Microsatellite genotypes were determined using the Fragment Analysis on CEQ 8000 (Beckman Coulter). Each fish was genotyped using a minimum of 14 microsatellite loci. Populations genotyped later in the work were genotyped using additional microsatellite loci that were developed during in the project from expressed sequence tag (EST) data (see table below).

Primers, PCR conditions and summary statistics for microsatellite loci used in this study.

| Primer codes (accession number) | PCR multiplex/ sequencer multiplex | Repeat (primers designed in this study) | Primer sequences  D2-D4 = Beckman Coulter code for fluorochrome for fluorescently-labelled primer. | Size Range (bp) | Number of alleles | Expected Heterozygosity | Reference |
| --- | --- | --- | --- | --- | --- | --- | --- |
|  |  |  |  |  |  |  |  |
| Rru2 | 1a/1 |  | D4 TTCCAGCTCAACTCTAAAGA  GCACCATGCAGTAACAAT | 88-120 | 13 | 0.7694 | [11] |
| Z21908 | 1a/1 |  | D4 ATTGATTAGGTCATTGCCCG  AGGAGTCATCGCTGGTGAGT | 131-189 | 15 | 0.6787 | http://zfin.org |
| CypG27 | 1a/1 |  | D2 AAGGTATTCTCCAGCATTTAT  GAGCCACCTGGAGACATTACT | 206-394 | 41 | 0.8154 | [12] |
| Ca3 | 1a/1 |  | D4 TCTAGCCCCCAAATTTTACGG  GGACAGTGAGGGACGCAGAC | 181-367 | 58 | 0.9526 | [13] |
| Ca1 | 1b/1 |  | AAGACGATGCTGGATGTTTAC  D3 CTATAGCTTATCCCGGCAGTA | 99-147 | 25 | 0.7885 | [13] |
| CypG24 | 1b/1 |  | D4 CTGCCGCATCAGAGATAAACACTT  TGGCGGTAAGGGTAGACCAC | 172-228 | 20 | 0.8302 | [12] |
| Rru044 (EG537044) | 1b/1 | [GT]^24^ | D2 ACATCAGGGATGTGCCTTCT  TTGGAAGCTGCAGTTGAAAT | 109-215 | 33 | 0.8902 | This study (EST) |
| CypG30 | 1c/1 |  | D3 GAAAAACCCTGAGAAATTCAAAAGA  GGACAGGTAAATGGATGAGGAGATA | 152-306 | 66 | 0.9505 | [12] |
| Rru4 | 2/2 |  | D4 TAAGCAGTGACCAGAATCCA  CAAAGCCTCAAAAGCACAA | 170-280 | 19 | 0.6617 | [11] |
| Lsou34 | 2/2 |  | D3 CCAGACAGGGTGATGATTCC  GTAGCGACGTTCAGGTCTCG | 219-235 | 8 | 0.5583 | [14], tested by [15] |
| LC290 | 2/2 |  | D3 CCCTAATGGCCCTCAATACA  ACTTCGCTGGCTTGACAAAT | 172-206 | 21 | 0.8111 | [16] |
| Llea029 | 2/2 |  | D4 TTTACCAGCATCCCCCAT  CATTTCACTCACTGAAGGAGAAC | 236-418 | 49 | 0.8846 | [15] |
| Llea131 | 2/2 |  | D4 TGACCTTTGCTATTTCAACATAACT  GTGTTTGAAATGTTTGTGCG | 92-136 | 21 | 0.7847 | [15] |
| N7K4 | 2/2 |  | ACGAGCATCAGTATCCAGAGACAC  D3 CATGTTTCCACATCTGAGCTAAAA | 115-171 | 12 | 0.5575 | [17], tested by [15] |
| Lsou08 | 2/2 |  | GCGGTGAACAGGCTTAACTC  D2 TAGGAACGAAGAGCCTGTGG | 171-223 | 18 | 0.6742 | [14], tested by [15] |
| Lid11 | 2/2 |  | D4 CTCCTGATTCTTTGTCTGACT  TTATTATTTCCTGTGGTGATTG | 178-386 | 25 | 0.8272 | [11] |
| Rru188 (EG542188) *^a, b^* | 2/2 | [AC]^34^[AG]^9^ | D3 TCGTTCATCAGTGCAACTGTC  TGAACAGAAAGTGTGAAGAAACAA | 233-383 | 66 | 0.9486 | This study (EST) |
| Rru710 (EG532710) *^a, b^* | 2/2 | [GA]^22^ | D3 AGTGGATTGGGCAGTATTGG  CTGAGGGATGTGGCTAATCA | 290-404 | 45 | 0.9056 | This study (EST) |
| Rru387 (EG534387) *^b^* | 2/2 | [GA]^24^ | D2 CTGCTCGTTTCTCACCATGA  TCAGGCACACAAACACATGA | 101-167 | 28 | 0.7974 | This study (EST) |
| Rru478 (EG545478) *^b^* | 2/2 | [GT]^16^ [GA]^1^ [GA]^5^ | D2 GGCTTCAGAAACCGTGAAAC  GTCTTCGGCCCAGTCTACAG | 308-346 | 16 | 0.7774 | This study (EST) |
| Rru288 (EG543288) *^b^* | 2/2 | [AT]^4^[AC]^19^ | D2 CTCATGCCCTCCGATACCTA  GGAATGCTCCAAATGTGTCA | 261-293 | 19 | 0.8121 | This study (EST) |

*^a^* Rru188 was replaced with Rru710 in multiplex 2.

*^b^* Primers designed in this study by searching expressed sequence tag (EST) sequences for dinucleotide repeats then designing primers flanking these regions using the program Primer3 [18].

***Population genetic analysis***

Methods used to calculate effective population sizes and to some extent analyse population genetic structure assume that loci are in Hardy-Weinberg equilibrium (HWE), are not in linkage, and are not in linkage with quantitative trait loci (QTL) that are under selection. As violations of these assumptions could result in inaccurate *N*_e_ estimates, tests were undertaken to identify loci that do not conform to these expectations and with genotyping errors in order to exclude these loci from further analyses.

Deviations from HWE for each locus in each population were calculated in GenAlEx 6 [19]. Possible linkage between microsatellite loci was investigated using GENEPOP ON THE WEB version 4.0.10 [20, 21], with critical levels of significance adjusted for multiple tests using the Bonferroni correction procedure [22] (i.e. for a population sample with 14 microsatellites genotyped, initial P = 0.05/14 = 0.0036). For each locus in each population sample Micro-Checker v.2.2.3 [23] was used to examine the presence of null alleles, allelic dropout and potential scoring errors caused by microsatellite stuttering. The program LOSITAN was used to identify loci that are potentially in linkage with QTL that are under selection [24]. Subsequently, the following loci were excluded from all analyses: CypG30 due to scoring problems in several populations and Rru188 and Rru044 as they significantly deviated from HWE in more than 7 of the 10 populations; Lsou08 was excluded from *N*_e_ and genetic bottleneck analyses as analysis suggested it was in linkage with a QTL under selection. Locus Ca3 was implicated in departure from HWE equilibrium in 5 of the 39 population samples, compared to a maximum of 3 for the other loci. There was no evidence of large allelic dropout or scoring errors caused by stuttering.

Less than 1.6% of all pairwise comparisons showed significant linkage disequilibrium after sequential Bonferroni corrections. No pairs of loci were found to be in linkage in more than 3 of the population samples, so linkage disequilibrium was deemed to be negligible. In the LeeHyd’95 sample, six of the 14 loci showed departure from HWE (compared to a maximum of 3 loci in other sites) and analysis in Microchecker [23] suggested the presence of null alleles in these six loci. This may have resulted from allelic dropout, due to the poor quality of DNA obtained from this sample, which had 7% missing data, compared to an average of 1.52% for the complete data set. No locus consistently showed evidence for null alleles.

***Population bottlenecks***

To test for evidence of recent population bottlenecks which may occur when actual or effective population sizes are substantially decreased for several generations, the microsatellite data were analysed using the program BOTTLENECK [25, 26]. This program tests for a relative excess in heterozygosity that is apparent for a few generations after a bottleneck event; this arises because allelic diversity declines faster than heterozygosity due to loss of rare alleles. Allele frequency distributions were compared to that of populations in mutation-drift equilibrium using the two-phase model (TPM, with a 70% stepwise mutation model). Deviations between observed and expected frequency distributions were tested using Wilcoxon’s signed rank test. BOTTLENECK was run for 10,000 iterations.

***Population trees***

Cavalli-Sforza and Edwards’ chord distance measure *D*_C_ [27] was used to construct trees, which has been shown to perform well in recovering the correct phylogeny in closely related populations [28]; the resulting tree was visualised using the Molecular Evolutionary Genetic Analysis (**MEGA 5)** program [29].

***Isolation with migration analysis***

The program IM_A_2 [30] simultaneously estimates gene flow, effective population sizes, and the time of splitting of the ancestral population. IM_A_2 implements a coalescent-based Markov chain Monte Carlo (MCMC) analysis method under an isolation-with-migration model of population divergence. IM_A_2 analysis is appropriate for populations where genetic equilibrium may not be valid, as may be the case with the roach populations analysed in this study. We used an estimated generation time of 4 years and a microsatellite mutation rate (*u*) of 0.0005 (0.00005, 0.005); microsatellite mutation rates (*u*) for other cyprinid fish have been estimated at 1.5 x 10^-4^ for zebrafish [31] and 5.6 x 10^-4^ for common carp (95% CI: 1.5 x 10^-4^ – 1.6 x 10^-3^) [32]. We assumed a stepwise mutation model. A series of prior runs were used to determine priors for subsequent runs. Final runs consisted of ten chains heated linearly, an initial burn-in of 1,000,000 steps and sampling every ten steps for over 25,000,000 generations.

***Principal component and STRUCTURE analyses***

**P**rincipal component analyses (PCA) based on Nei’s *D*_A_ genetic distances [33], were carried out in GenAlEx [19]. To estimate the number of genetically differentiated clusters (*K*) within the data set, the **program** STRUCTURE v2.3.3 was used; this assigns individuals to probable clusters using a Bayesian approach [34]. We used the standard model and a “*locprior*” model designed to detect weak population genetic structure by using sampling location information. Log-likelihood values for each *K* (ranging from 1 – 42) were computed from multi-locus genotypes by running STRUCTURE three times with 125,000 repetitions each time (burn-in = 100,000 iterations). The most likely number of populations (*K*) was evaluated visually and with the method of Evanno et al. [35], as implemented by STRUCTURE Harvester [36].

***Effective population size***

The two single sample methods employed use different aspects of the microsatellite data. Previous studies have shown estimates from these techniques to be positively correlated with each other and to be less variable than *N*_e (LD)_, the other commonly used single-sample technique [37]. ONeSAMP has been found to provide the most precise estimates of *N*_e_ from the single-sample methods [38]. It is also predicted to perform well with overlapping generations using random samples of reproductively mature adults [39]. For each population, we performed analysis with a Bayesian prior range of 2-500. *N*_e_ calculated using the ONeSAMP method is usually heavily influenced by linkage disequilibrium.

*N*_e_ was also estimated using the sibship assignment method in the program COLONY v2.0 [40], which uses the frequencies of full- and half-siblings in the sample to estimate *N*_e_. The method has been shown to perform well both with simulated and empirical data [38], although it appears to be most accurate with a random sample of individuals belonging to a single cohort, which is not the case in our study.

In order to calculate error rates for each locus, as required by COLONY, 136 fish were genotyped twice, giving error rates of: LC290 = 0.02; Lea029 = 0.03; Rru478 = 0.02; Ca3 = 0.02; CypG24 = 0.02, CypG27 = 0.02 and Z21908 = 0.02. No errors were observed for the other loci. Long runs were used with the full likelihood method, assuming nonrandom mating. As inclusion of potential parents in the sample results in downwardly biased estimates of *N*_b_, because parent-offspring dyads are likely to be inferred as full-sibs, sibships that involved fish that differed in age by more than 3 years were excluded. As roach populations have overlapping generations, a sample taken at random from the population contains individuals at different life stages, in which case COLONY estimates the effective number of breeders (*N*_b_) which, like *N*_e_, is influenced by sex ratio, variance in reproductive successes of males and females, but does not consider random processes involving multiple life stages. Nevertheless, for simplicity we refer to estimates from both ONeSAMP and COLONY as *N*_e_.

Calculations of *N*_e_ using the temporal method were made using the software TempoFs [41] following either ‘sample plan 1’, if individuals were sampled non-destructively, or ‘sample plan 2’, when individuals were sampled destructively before reproduction and were not returned to the population. This method generally performs better than other temporal methods if allele frequencies are skewed, as is common in microsatellite data [41]. We used a mean generation time of 4 years, although in reality generation time may vary between sample sites. In addition, *N*_e_ was calculated using the classical moment-based method of Waples [42], using NeEstimator [43] to allow comparison with other studies.

***Statistical analysis***

A generalized linear model (GLM) was used to explore the relationship between *N*_e(ABC)_ and other variables measured in this study. Our analyses focused on *N*_e(ABC)_ as the response variable, as this variable has been shown to perform well in other studies [37, 38]. Furthermore, the influence of restocking that occurred prior to 2000 would have minimal influence on *N*_e(ABC)_, which is influenced primarily by breeding patterns in the parental generation, in contrast to measures of genetic diversity and population bottlenecks, which are influenced by demography over many generations. We excluded population samples where fewer than 30 fish had been sampled and those where recent restocking had occurred (AirDar, WanMoh). Averages were used for sites sampled at multiple time points. This dataset included 28 sample sites, all of which were separated by physical barriers. Geographic/phylogenetic group (Kennet, Arun, Nene, Lee & Stort, rest of the Thames, Blackwater & Chelmer, Trent) was also included as a covariate to control (to some extent) for differences between regions, and average flow rate was included to control for river size. We also analyzed data for a subset of sample sites (19 sites) where roach density (calculated from 2000 onwards) had been estimated using the same methodology. Densities were obtained from the National Fish Populations Database (NFPD) held by the Environment Agency, UK. Additionally, to investigate the apparent reduction in variance with increased estrogen concentration, data were initially split into 100 bins and an algorithm was used to increase bin size until each bin had at least 5 data points in each, sufficient for random resampling. Bootstrapping was then used to estimate the standard deviation within each bin.

***Genetic diversity statistics***

Differences in allelic richness, expected heterozygosity and observed heterozygosity among samples were tested using ANOVA in R.

**References**

1. Williams RJ, Keller VDJ, Johnson AC, Young AR, Holmes MGR, Wells C, Gross-Sorokin M, Benstead R: **A national risk assessment for intersex in fish arising from steroid estrogens.** *Environ Toxicol Chem* 2009, **28:**220-230.

2. Williams RJ, Churchley JH, Kanda R, Johnson AC: **Comparing predicted against measured steroid estrogen concentrations and the associated risk in two United Kingdom river catchments.** *Environ Toxicol Chem* 2012, **31:**892-898.

3. Jobling S, Williams R, Johnson A, Taylor A, Gross-Sorokin M, Nolan M, Tyler CR, van Aerle R, Santos E, Brighty G: **Predicted exposures to steroid estrogens in UK rivers correlate with widespread sexual disruption in wild fish populations.** *Environ Health Persp* 2006, **114:**32-39.

4. Jobling S, Nolan M, Tyler CR, Brighty G, Sumpter JP: **Widespread sexual disruption in wild fish.** *Environ Sci Technol* 1998, **32:**2498-2506.

5. Jobling S, Coey S, Whitmore JG, Kime DE, Van Look KJW, McAllister BG, Beresford N, Henshaw AC, Brighty G, Tyler CR, Sumpter JP: **Wild intersex roach (*Rutilus rutilus*) have reduced fertility.** *Biol Reprod* 2002, **67:**515-524.

6. Harris CA, Hamilton PB, Runnalls TJ, Vinciotti V, Henshaw A, Hodgson D, Coe TS, Jobling S, Tyler CR, Sumpter JP: **The consequences of feminization in breeding groups of wild fish.** *Environ Health Persp* 2011, **119:**306-311.

7. Estoup A, Largiader CR, Perrot E, Chourrout D: **Rapid one-tube DNA extraction for reliable PCR detection of fish polymorphic markers and transgenes.** *Mol Mar Biol Biotech* 1996, **5:**295-298.

8. Truett GE, Heeger P, Mynatt RL, Truett AA, Walker JA, Warman ML: **Preparation of PCR-quality mouse genomic DNA with hot sodium hydroxide and tris (HotSHOT).** *Biotechniques* 2000, **29:**52-+.

9. Wyatt PMW, Pitts CS, Butlin RK: **A molecular approach to detect hybridization between bream *Abramis brama*, roach *Rutilus rutilus* and rudd *Scardinius erythrophthalmus*.** *J Fish Biol* 2006, **69:**52-71.

10. Hamilton PB, Tyler CR: **Identification of microsatellite loci for parentage analysis in roach *Rutilus rutilus* and 8 other cyprinid fish by cross-species amplification, and a novel test for detecting hybrids between roach and other cyprinids.** *Mol Ecol Resour* 2008, **8:**462-465.

11. Barinova A, Yadrenkina E, Nakajima M, Taniguchi N: **Identification and characterization of microsatellite DNA markers developed in ide *Leuciscus idus* and Siberian roach *Rutilus rutilus*.** *Mol Ecol Notes* 2004, **4:**86-88.

12. Baerwald MR, May B: **Characterization of microsatellite loci for five members of the minnow family Cyprinidae found in the Sacramento-San Joaquin Delta and its tributaries.** *Mol Ecol Notes* 2004, **4:**385-390.

13. Dimsoski P, Toth GP, Bagley MJ: **Microsatellite characterization in central stoneroller *Campostoma anomalum* (Pisces: Cyprinidae).** *Mol Ecol* 2000, **9:**2187-2189.

14. Muenzel FM, Sanetra M, Salzburger W, Meyer A: **Microsatellites from the vairone *Leuciscus souffia* (Pisces: Cyprinidae) and their application to closely related species.** *Mol Ecol Notes* 2007, **7:**1048-1050.

15. Dubut V, Sinama M, Martin J-F, Meglecz E, Fernandez J, Chappaz R, Gilles A, Costedoat C: **Cross-species amplification of 41 microsatellites in European cyprinids: A tool for evolutionary, population genetics and hybridization studies.** *BMC Res Notes* 2010, **3:**135.

16. Vyskocilova M, Simkova A, Martin JF: **Isolation and characterization of microsatellites in *Leuciscus cephalus* (Cypriniformes, Cyprinidae) and cross-species amplification within the family Cyprinidae.** *Mol Ecol Notes* 2007, **7:**1150-1154.

17. Mesquita N, Cunha C, Hanfling B, Carvalho GR, Ze-Ze L, Tenreiro R, Coelho MM: **Isolation and characterization of polymorphic microsatellite loci in the endangered Portuguese freshwater fish *Squalius aradensis* (Cyprinidae).** *Mol Ecol Notes* 2003, **3:**572-574.

18. Rozen S, Skaletsky HJ: **Primer3 on the WWW for general users and for biologist programmers.** In *Bioinformatics Methods and Protocols: Methods in Molecular Biology.* Edited by Krawetz S, Misener S. Totowa, NJ: Humana Press; 2000: 365-386

19. Peakall R, Smouse PE: **GenAlEx 6: genetic analysis in Excel. Population genetic software for teaching and research.** *Mol Ecol Notes* 2006, **6:**288-295.

20. Raymond M, Rousset F: **Genepop (Version-1.2) - Population-genetics software for exact tests and ecumenicism.** *J Hered* 1995, **86:**248-249.

21. Rousset F: **GENEPOP ' 007: a complete re-implementation of the GENEPOP software for Windows and Linux.** *Mol Ecol Resour* 2008, **8:**103-106.

22. Rice WR: **Analyzing tables of statistical tests.** *Evolution* 1989, **43:**223-225.

23. Van Oosterhout C, Hutchinson WF, Wills DPM, Shipley P: **MICRO-CHECKER: software for identifying and correcting genotyping errors in microsatellite data.** *Mol Ecol Notes* 2004, **4:**535-538.

24. Antao T, Lopes A, Lopes RJ, Beja-Pereira A, Luikart G: **LOSITAN: A workbench to detect molecular adaptation based on a F(st)-outlier method.** *BMC Bioinformatics* 2008, **9**.

25. Cornuet JM, Luikart G: **Description and power analysis of two tests for detecting recent population bottlenecks from allele frequency data.** *Genetics* 1996, **144:**2001-2014.

26. Piry S, Luikart G, Cornuet JM: **BOTTLENECK: A computer program for detecting recent reductions in the effective population size using allele frequency data.** *J Hered* 1999, **90:**502-503.

27. Cavalli-Sforza L, Edwards A: **Phylogenetic analysis: models and estimation procedures.** *Am J Hum Genet* 1967, **19:**233-257.

28. Goldstein DB, Pollock DD: **Launching microsatellites: A review of mutation processes and methods of phylogenetic inference.** *J Hered* 1997, **88:**335-342.

29. Tamura K, Peterson D, Peterson N, Stecher G, Nei M, Kumar S: **MEGA5: Molecular Evolutionary Genetics Analysis using Maximum Likelihood, Evolutionary Distance, and Maximum Parsimony Methods.** *Mol Biol Evol* 2011.

30. Hey J: **Isolation with migration models for more than two populations.** *Mol Biol Evol* 2010, **27:**905-920.

31. Shimoda N, Knapik EW, Ziniti J, Sim C, Yamada E, Kaplan S, Jackson D, de Sauvage F, Jacob H, Fishman MC: **Zebrafish genetic map with 2000 microsatellite markers.** *Genomics* 1999, **58:**219-232.

32. Yue GH, David L, Orban L: **Mutation rate and pattern of microsatellites in common carp (*Cyprinus carpio* L.).** *Genetica* 2007, **129:**329-331.

33. Nei M, Tajima F, Tateno Y: **Accuracy of estimated phylogenetic trees from molecular-data.2. Gene-Frequency Data.** *J Mol Evol* 1983, **19:**153-170.

34. Pritchard JK, Stephens M, Donnelly P: **Inference of population structure using multilocus genotype data.** *Genetics* 2000, **155:**945-959.

35. Evanno G, Regnaut S, Goudet J: **Detecting the number of clusters of individuals using the software STRUCTURE: a simulation study.** *Mol Ecol* 2005, **14:**2611-2620.

36. Earl DA: **Structure Harvester v0.3.** Available from http://www.users.soe.ucsc.edu/~dearl/software/struct_harvest/; 2009.

37. Phillipsen IC, Funk WC, Hoffman EA, Monsen KJ, Blouin MS: **Comparative analyses of effective population size within and among species: ranid frogs as a case study.** *Evolution* 2011, **65:**2927-2945.

38. Barker JSF: **Effective population size of natural populations of *Drosophila buzzatii*, with a comparative evaluation of nine methods of estimation.** *Mol Ecol* 2011, **20:**4452-4471.

39. Robinson JD, Moyer GR: **Linkage disequilibrium and effective population size when generations overlap.** *Evol Appl* 2012**:**n/a.

40. Jones OR, Wang J: **COLONY: a program for parentage and sibship inference from multilocus genotype data.** *Mol Ecol Resour* 2009, **10:**551-555.

41. Jorde PE, Ryman N: **Unbiased estimator for genetic drift and effective population size.** *Genetics* 2007, **177:**927-935.

42. Waples RS: **A generalized approach for estimating effective population-size from temporal changes in allele frequency.** *Genetics* 1989, **121:**379-391.

43. Peel D, Ovenden JR, Peel SL: **NEESTIMATOR: Software for Estimating Effective Population Size.** 1.3 edition: Queensland Government: Department of Primary Industries and Fisheries, Brisbane, Queensland; 2004.
