# Supplementary material for: Populations of a cyprinid fish are self-sustaining despite widespread feminization of males
Source: BMC Biol. 2014 Jan 13;12:1. doi: 10.1186/1741-7007-12-1 (PMC3922797; doi:10.1186/1741-7007-12-1)
Supplement: Additional file 3 — Graph showing statistical differences in genetic diversity among roach Rutilus rutilus populations. [file 1741-7007-12-1-S3.ppt]

## Slide 1
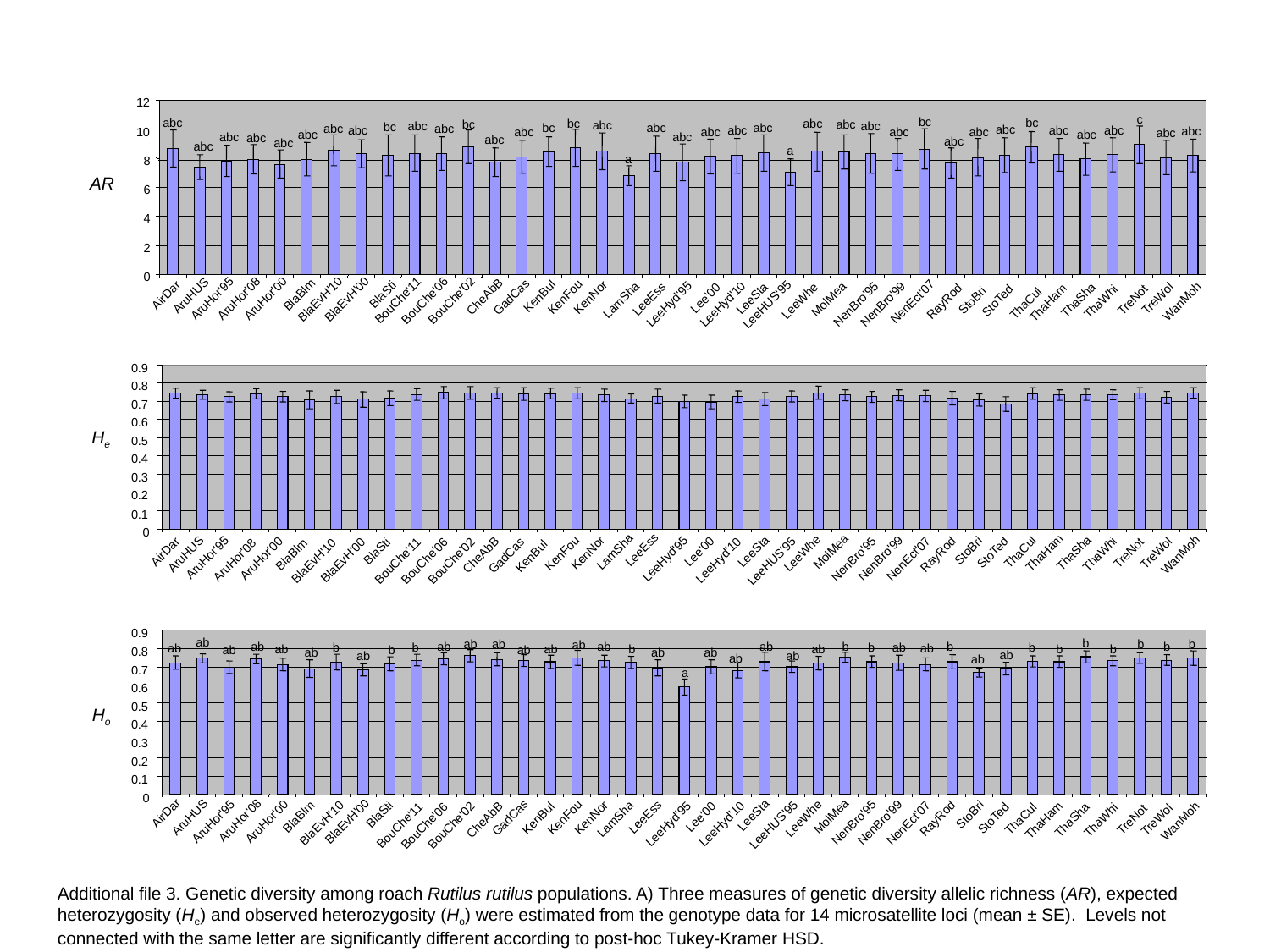

12
c
bc
abc
bc
bc
abc
bc
abc
abc
abc
abc
bc
bc
abc
abc
abc
abc
abc
abc
abc
abc
abc
abc
abc
abc
abc
abc
abc
abc
abc
abc
abc
10
abc
abc
abc
abc
abc
a
a
8
AR
6
4
2
0
BlaSti
BlaBlm
AirDar
AruHUS
KenBul
CheAbB
GadCas
KenFou
KenNor
Lee'00
LeeSta
TreWol
AruHor'95
AruHor'08
AruHor'00
BlaEvH'00
BlaEvH'10
LeeEss
MolMea
TreNot
LamSha
ThaSha
StoBri
BouChe'11
BouChe'06
BouChe'02
LeeWhe
StoTed
WanMoh
RayRod
NenEct'07
ThaWhi
ThaHam
ThaCul
NenBro'95
NenBro'99
LeeHyd'95
LeeHyd'10
LeeHUS'95
0.9
0.8
0.7
0.6
He
0.5
0.4
0.3
0.2
0.1
0
LeeEss
StoBri
BlaSti
Lee'00
LeeSta
AirDar
LamSha
MolMea
ThaCul
StoTed
ThaHam
TreNot
KenNor
TreWol
ThaSha
LeeWhe
KenFou
AruHUS
KenBul
ThaWhi
RayRod
WanMoh
BlaBlm
CheAbB
GadCas
AruHor'95
NenBro'99
AruHor'00
NenEct'07
LeeHyd'95
NenBro'95
AruHor'08
LeeHyd'10
BlaEvH'00
BlaEvH'10
BouChe'11
BouChe'06
BouChe'02
LeeHUS'95
0.9
ab
b
ab
ab
b
b
ab
ab
b
b
ab
ab
ab
b
b
b
b
ab
b
ab
ab
ab
ab
ab
b
b
b
ab
ab
b
ab
ab
ab
ab
ab
ab
0.8
ab
ab
a
0.7
0.6
Ho
0.5
0.4
0.3
0.2
0.1
0
AirDar
BlaSti
StoBri
LeeSta
MolMea
BlaBlm
Lee'00
LeeEss
AruHUS
KenFou
GadCas
KenNor
KenBul
StoTed
LeeWhe
RayRod
ThaCul
LamSha
TreNot
TreWol
ThaSha
CheAbB
ThaHam
ThaWhi
AruHor'08
WanMoh
BlaEvH'00
AruHor'95
AruHor'00
NenBro'95
NenBro'99
NenEct'07
BlaEvH'10
LeeHyd'95
LeeHyd'10
LeeHUS'95
BouChe'11
BouChe'02
BouChe'06
Additional file 3. Genetic diversity among roach Rutilus rutilus populations. A) Three measures of genetic diversity allelic richness (AR), expected heterozygosity (He) and observed heterozygosity (Ho) were estimated from the genotype data for 14 microsatellite loci (mean ± SE). Levels not connected with the same letter are significantly different according to post-hoc Tukey-Kramer HSD.
