# Supplementary material for: Populations of a cyprinid fish are self-sustaining despite widespread feminization of males
Source: BMC Biol. 2014 Jan 13;12:1. doi: 10.1186/1741-7007-12-1 (PMC3922797; doi:10.1186/1741-7007-12-1)
Supplement: Additional file 5 — Multidimensional scaling plots of pairwise D A distances [[72]]. [file 1741-7007-12-1-S5.ppt]

## Slide 1
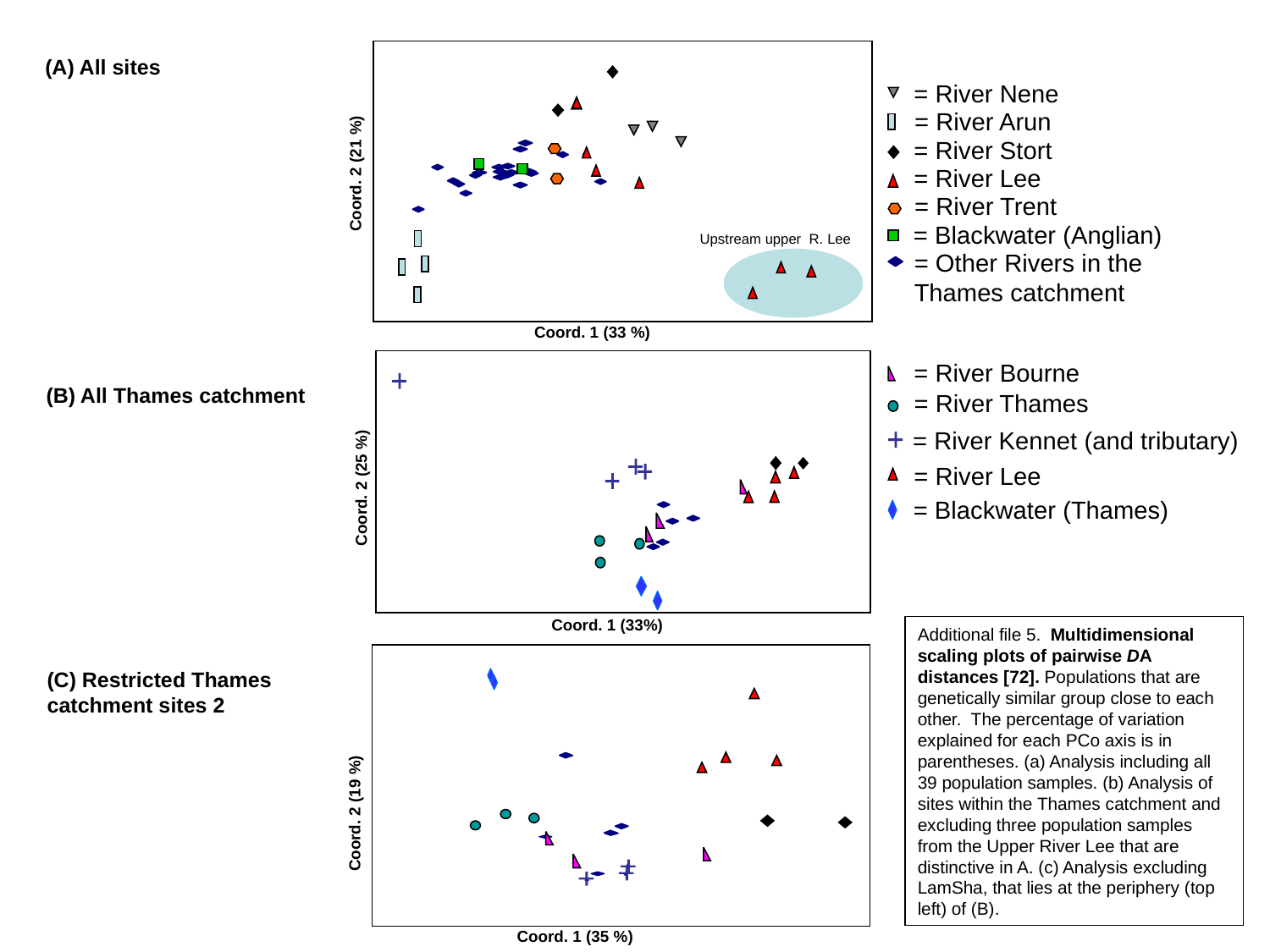

(A) All sites
= River Nene
= River Arun
Coord. 2 (21 %)
= River Stort
= River Lee
= River Trent
= Blackwater (Anglian)
Upstream upper R. Lee
= Other Rivers in the
Thames catchment
Coord. 1 (33 %)
= River Bourne
= River Thames
(B) All Thames catchment
= River Kennet (and tributary)
Coord. 2 (25 %)
= River Lee
= Blackwater (Thames)
Coord. 1 (33%)
Additional file 5. Multidimensional scaling plots of pairwise DA distances [72]. Populations that are genetically similar group close to each other. The percentage of variation explained for each PCo axis is in parentheses. (a) Analysis including all 39 population samples. (b) Analysis of sites within the Thames catchment and excluding three population samples from the Upper River Lee that are distinctive in A. (c) Analysis excluding LamSha, that lies at the periphery (top left) of (B).
(C) Restricted Thames catchment sites 2
Coord. 2 (19 %)
Coord. 1 (35 %)
