# Supplementary material for: Populations of a cyprinid fish are self-sustaining despite widespread feminization of males
Source: BMC Biol. 2014 Jan 13;12:1. doi: 10.1186/1741-7007-12-1 (PMC3922797; doi:10.1186/1741-7007-12-1)
Supplement: Additional file 6 — Structure analyses plots using the locprior model and analysis of optimum number of genetic units in Structure Harvester [[73]]. [file 1741-7007-12-1-S6.ppt]

## Slide 1
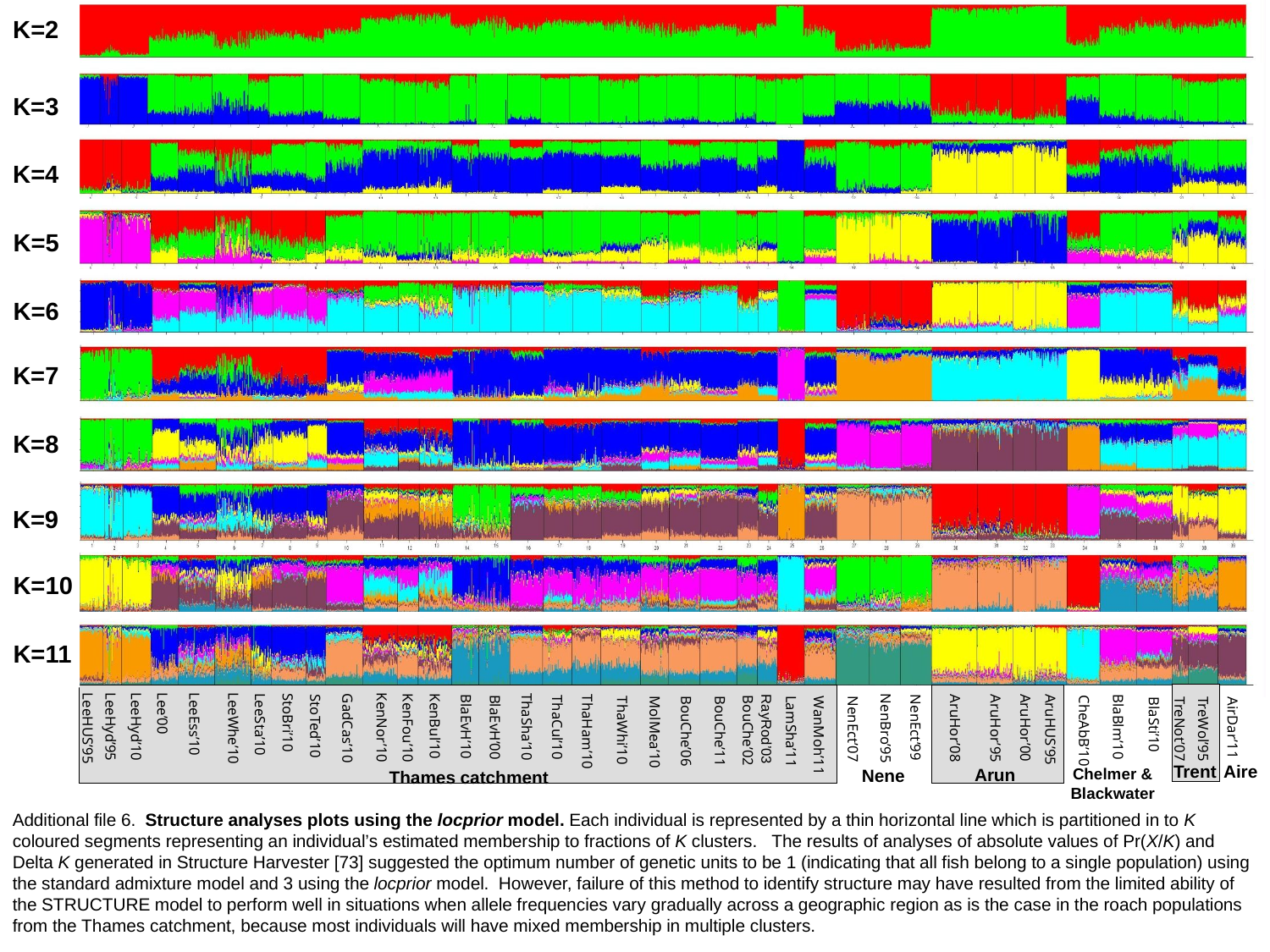

K=2
K=3
K=4
K=5
K=6
K=7
K=8
K=9
K=10
K=11
LeeHyd’95
LeeHyd’10
LeeWhe’10
LeeEss’10
Lee’00
LeeHUS’95
KenNor’10
LeeSta’10
StoBri’10
ThaSha’10
GadCas’10
KenBul’10
NenBro’95
KenFou’10
AruHor’00
AruHUS’95
BlaBlm’10
StoTed’10
BlaEvH’10
AruHor’08
AruHor’95
ThaHam’10
RayRod’03
NenEct’99
BlaEvH’00
CheAbB’10
ThaCul’10
BouChe’02
LamSha’11
BouChe’11
MolMea’10
BouChe’06
NenEct’07
WanMoh’11
ThaWhi’10
BlaSti’10
TreWol’95
AirDar’11
TreNot’07
Trent
Aire
Arun
 Chelmer &
Blackwater
Nene
Thames catchment
Additional file 6. Structure analyses plots using the locprior model. Each individual is represented by a thin horizontal line which is partitioned in to K coloured segments representing an individual’s estimated membership to fractions of K clusters. The results of analyses of absolute values of Pr(X/K) and Delta K generated in Structure Harvester [73] suggested the optimum number of genetic units to be 1 (indicating that all fish belong to a single population) using the standard admixture model and 3 using the locprior model. However, failure of this method to identify structure may have resulted from the limited ability of the STRUCTURE model to perform well in situations when allele frequencies vary gradually across a geographic region as is the case in the roach populations from the Thames catchment, because most individuals will have mixed membership in multiple clusters.
