# Supplementary material for: Populations of a cyprinid fish are self-sustaining despite widespread feminization of males
Source: BMC Biol. 2014 Jan 13;12:1. doi: 10.1186/1741-7007-12-1 (PMC3922797; doi:10.1186/1741-7007-12-1)
Supplement: Additional file 7 — Correlation between genetic distance and geographic distance (km) between pairs of sites for 24 population samples from the Thames catchment. [file 1741-7007-12-1-S7.ppt]

## Slide 1
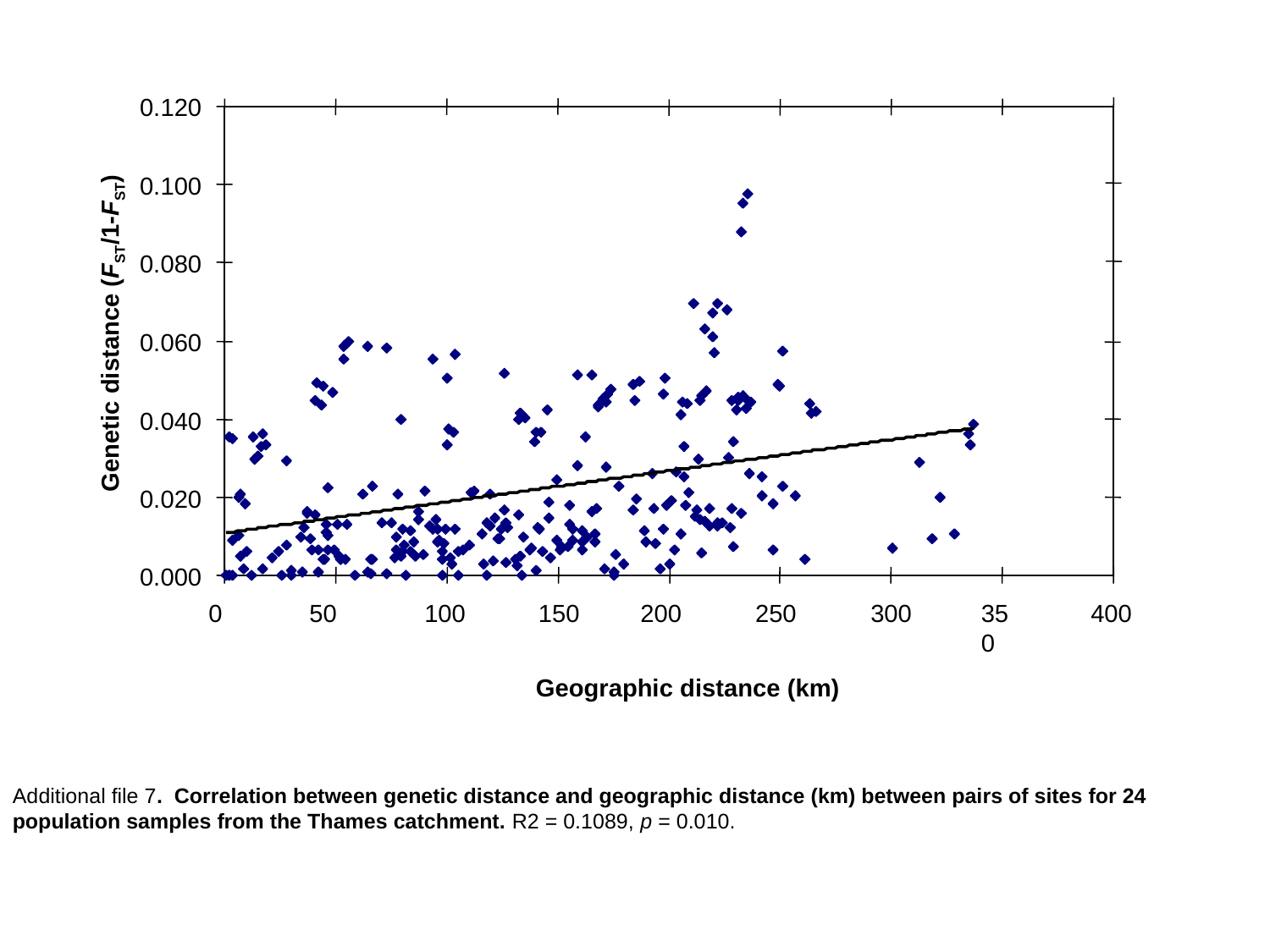

0.120
0.100
Genetic distance (FST/1-FST)
0.080
0.060
0.040
0.020
0.000
0
50
100
150
200
250
300
350
400
Geographic distance (km)
Additional file 7. Correlation between genetic distance and geographic distance (km) between pairs of sites for 24 population samples from the Thames catchment. R2 = 0.1089, p = 0.010.
